# Common cuckoo eggs are more resistant to puncture by the host

**DOI:** 10.1016/j.ijppaw.2024.101003

**Published:** 2024-10-16

**Authors:** Hanlin Yan, Longwu Wang, Wei Liang

**Affiliations:** a Ministry of Education Key Laboratory for Ecology of Tropical Islands, Key Laboratory of Tropical Animal and Plant Ecology of Hainan Province, College of Life Sciences, Hainan Normal University, Haikou, 571158, China; b School of Life Sciences, Guizhou Normal University, Guiyang, 550001, China

**Keywords:** Brood parasitism, Common cuckoo, Oriental reed warbler, Eggshell, Host, Puncture-ejection

## Abstract

The puncture resistance hypothesis suggests that thick-shelled eggs of parasitic birds can resist puncture-ejection by the host. However, few experiments have yet been conducted to test this hypothesis in terms of natural host behavior (e.g., pecking at foreign eggs). To explore whether the eggshells of common cuckoos (*Cuculus canorus*) are resistant to puncture-ejection by their common hosts, Oriental reed warblers (*Acrocephalus orientalis*), we designed experiments to investigate if and how breeding Oriental reed warblers peck at foreign eggs that includes common cuckoo, Oriental reed warbler and budgerigar (*Melopsittacus undulatus*) eggs. The results showed that, given the same frequency of egg pecking, the probability of eggshell breakage was 87.5% for eggs of the Oriental reed warbler and 0% for eggs of the common cuckoo, with a significant difference (*P* = 0.001, Fisher's exact test). Our study shows clearly that common cuckoos' eggshells are less susceptible to puncture-ejection than those of Oriental reed warblers and budgerigars. This indicates that the eggshells of common cuckoos can resist host Oriental reed warblers' puncture-ejection, supporting the puncture resistance hypothesis.

## Highlights

• Eggshells of common cuckoos (*Cuculus canorus*) are less susceptible to puncture-ejection than those of Oriental reed warbler (*Acrocephalus orientalis*) hosts and budgerigars (*Melopsittacus undulatus*).
• We provided experimental evidence for the puncture resistance hypothesis showing that cuckoo eggshells can resist hosts' puncture-ejection.

## Graphical abstract

This experimental study showed that eggshells of common cuckoos (*Cuculus canorus*) are less susceptible to puncture-ejection than those of Oriental reed warbler (*Acrocephalus orientalis*) hosts and budgerigars (*Melopsittacus undulatus*).

**Figure:**
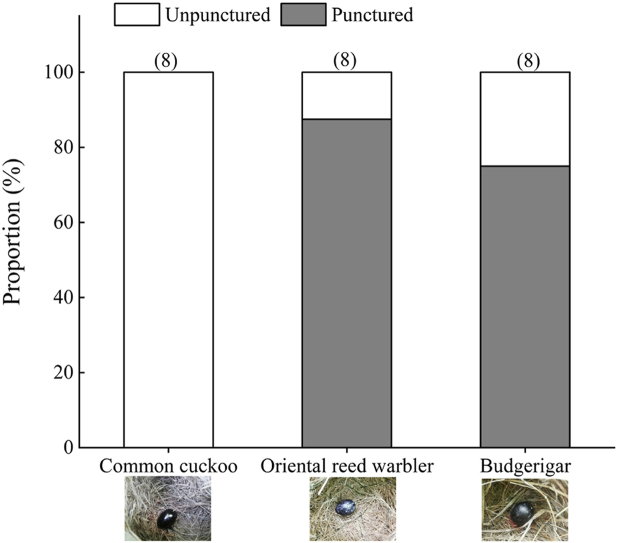

## 1 Introduction

Avian brood parasitism is a behavior where a bird does not construct its own nest but lays its eggs in the nests of other birds (hosts) (Davies 2000). Thus, this shifts the reproductive costs of incubation and brood rearing onto the host (Payne 1977). Successful brood parasitism prompts the host to evolve a range of antiparasitic strategies (Davies 2000; Soler 2014), and egg rejection behavior is one of the most prevalent antiparasitic strategies in avian brood parasitism hosts (Davies 2011; Soler 2014). This represents a specific adaptive response by a host to parasitism (Langmore et al., 2005).

In turn, the parasitic have evolved a number of strategies to deal with their hosts. such as the thick eggshells are an adaptation to brood parasitism (López et al., 2018, 2023), and some researchers proposed many hypotheses in which thick eggshells can bring multiple benefits to the parasite (Antonov et al., 2012; Narushin et al., 2024). One of the main hypotheses is the puncture resistance hypothesis, which posits that the thick eggshell of the parasite can be used to resist puncture-ejection by the host (Swynnerton 1918; Spaw and Rohwer 1987; but see Holleley et al., 2022). This indicates that, for the hosts of brood parasites, those with small beaks are unable to grasp and throw away the eggs laid by the parasite using their beaks. Therefore, they must first pierce the eggshell with their beaks and then discard it outside the nest (Sealy 1996). However, only Sealy (1996) reported that warbling vireos (*Vireo gilvus*) rejected eggs of the brown-headed cowbird (*Molothrus ater*) and found that a small host could puncture or break the eggs and then remove or discard them. To some extent, this hypothesis has been tested. However, in the case of common cuckoos (*Cuculus canorus*), the most studied species in China, no experimental cases have been tested so far.

Oriental reed warblers (*Acrocephalus orientalis*) are among the most commonly used hosts of common cuckoos, and their interactions have reached an advanced stage of coevolution (Li et al., 2015, 2016, 2020; Ma et al., 2018; Ma and Liang 2021; Wang et al., 2022). Common cuckoos have developed a highly mimetic reed warbler clade in the corresponding study area (Wang et al., 2024), thus contributing to the fact that Oriental reed warblers have evolved a high level of egg recognition (Li et al., 2016, 2020; Yang et al., 2016, 2017). Oriental reed warblers peck through the eggshell when throwing away foreign eggs (with the habit of pecking at foreign eggs), pick up the eggs with the beak of their mouths through the pecked holes, and throw them out of the nest (Yang et al., 2016; Ma and Liang 2021). Therefore, are common cuckoo eggs resistant to puncture-ejection by their Oriental reed warbler hosts?

In this study, we designed experiments to observe the pecking of foreign eggs by Oriental reed warblers. The aim is to provide the experimental evidence to verify whether the eggshell thickness of common cuckoo eggs can resist the puncture-ejection of Oriental reed warblers. We expected that eggs of the common cuckoo would be less susceptible to pecking by Oriental reed warblers than host own eggs. In other words, the eggshells of common cuckoo eggs would be more resistant to puncture-ejection by host Oriental reed warblers than the eggshells of host own eggs.

## 2 Materials and methods

### 2.1 Study area and study species

The study site is at Sifangtuozi Farm (46°00′-46°22′ N, 123°46′-123°57′ E), which is located in Jilin Province, Northeast China. Every year during the breeding season (June–August), large numbers of Oriental reed warblers migrate to the reed beds surrounding agricultural ridges, diversion canals, ditches, and ponds in the region to breed (Yan et al., 2024). The Oriental reed warbler is one of the main hosts of common cuckoos, with a parasitism rate of 34.3–65.5% (Wang et al., 2020, 2021, 2022; Yang et al., 2014, 2016, 2017). From June to August 2022, we conducted relevant field experiments in the study area.

### 2.2 Experimental design

In the study area, farmers often cut part of the reeds on the ridges for cultivation. Heavy rain and wind can cause many reeds to fall over, which can lead to the abandonment of nests by Oriental reed warblers that had previously nested in these areas; however, the warblers would re-select other areas for nesting and breeding. Therefore, we collected these abandoned nests and eggs (including eggs of parasitized common cuckoos already in the nest) from Oriental reed warblers during the breeding season (Yan et al., 2024). We reconditioned and reinforced the collected nests and measured the collected Oriental reed warbler eggs (egg length: 21.87 ± 0.92 mm, egg width: 15.87 ± 0.64 mm, egg weight: 2.88 ± 0.27 g, n = 16) and common cuckoo eggs (egg length: 22.33 ± 0.99 mm, egg width: 17.01 ± 0.52 mm, egg weight: 3.64 ± 0.37 g, n = 12). In addition, we tested budgerigar (*Melopsittacus undulatus*) eggs in our experiments, that were white unfertilized eggs purchased from the market for breeding, and these egg parameters were measured (egg length: 20.08 ± 0.90 mm, egg width: 15.31 ± 0.61 mm, egg weight: 2.03 ± 0.16 g, n = 25).

**Fig. 1:**
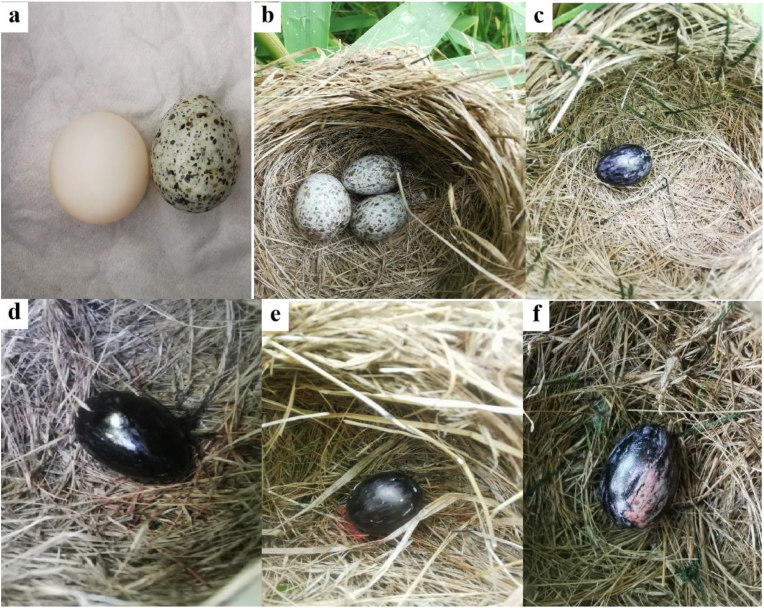
Different types of eggs. (a) Refers to the budgerigar egg and Oriental reed warbler egg, from left to right; (b) refers to the common cuckoo egg and two Oriental reed warbler eggs, from left to right; (c) refers to the blacked Oriental reed warbler egg; (d) refers to the blacked common cuckoo egg; (e) refers to the blacked budgerigar egg; and (f) refers to the blacked common cuckoo that were not punctured by the Oriental reed warbler.

To minimize interference with the natural reproduction of Oriental reed warblers, we chose to add a new, artificially fixed nest near their nest (around 50 cm in diameter) during the brooding stage. We placed a single Oriental reed warbler, common cuckoo, or budgerigar egg (Fig. 1), as numerous studies have confirmed that hosts use degree of egg mimicry to reject eggs (Honza and Cherry 2017; Šulc et al., 2016); spots help Oriental reed warblers recognize eggs and egg size has less of an effect relative to other visual features of the eggshell (Li et al., 2020). The Oriental reed warbler can accurately recognize its own eggs (Ma and Liang 2021), so we chose to paint all three types of eggs black to hide the spots on the eggs of common cuckoos and the eggs of Oriental reed warblers. We tested each nest with these three types of eggs, and each time, we randomly selected one type of egg for the experiment. All eggs (including those that were not pecked through their shells by the Oriental reed warbler during the experiment) were not reused, and each nest was considered completed only after all three types of eggs had been tested. We installed a video recorder above the bird nest (Ou Chuang A8; Xiamen Shangyu Huajin Electronic Technology Co., Ltd. Xiamen, China) to record Oriental reed warblers pecking at three types of eggs.

### 2.3 Statistical analyses

We conducted statistical analysis on the video recordings of the egg-pecking experiments (based on the length of the video recordings within 30 min, if some videos were missing, the actual length of the video recordings would be used as the base) for the egg of each species at each nest. The main parameters included: number of egg pecks (the total number of effective pecks by Oriental reed warblers that either broke or did not break the eggs within 30 min), egg-pecking duration (the effective duration of pecking, with durations less than 1 s recorded as 1 s; resting time during pecking breaks was not included), frequency (the ratio of effective number of egg pecks to effective egg-pecking duration), and pecking outcome (whether the eggs were broken by Oriental reed warblers; 1 = broken eggshell, 0 = not broken). Detailed parameters are provided in the table (Data Table S1). Finally, we only focus on the analysis and discussion of “egg-pecking outcome,” “number of egg pecks,” “egg-pecking duration,” and “frequency”. The other parameters are presented in the data table only as general results (Data Table S1).

Fisher's exact test was used to compare the “egg-pecking outcome” (1 = broken eggshell, 0 = not broken) of Oriental reed warblers pecks three types of eggs (blacked-Oriental reed warbler vs. blacked-common cuckoo vs. blacked-budgerigar). Kruskal-Wallis test was used to compare “number of egg pecks,” “egg-pecking duration,” and “frequency,” of Oriental reed warblers pecks three types of eggs (blacked-Oriental reed warbler vs. blacked-common cuckoo vs. blacked-budgerigar) (Data Table S2). We analyzed the data using IBM SPSS Statistics 22.0 (IBM Inc., Armonk, NY, USA), Origin2022 (Origin Lab., Northampton, MA, USA) for graphing. All reported tests were two-tailed and *P* < 0.05 indicates statistical significance. Data were presented in the form of the mean ± standard deviation.

## 3 Results

We tested a total of 10 nests of Oriental reed warblers in pecking experiments on three types of eggs (blacked-Oriental reed warblers, blacked-common cuckoos, and blacked-budgerigars). However, in one nest, Oriental reed warblers did not visit during the testing period, and in another nest, they visited but did not peck any of the three types of eggs. Therefore, the parameters for these two nests were not included in the analysis. Additionally, the video file for one nest where Oriental reed warblers broke the eggshell was damaged. However, upon nest inspection, we found remnants of broken eggshells and egg fluid, so we only recorded the outcome as a broken eggshell without recording other parameters.

**Fig. 2:**
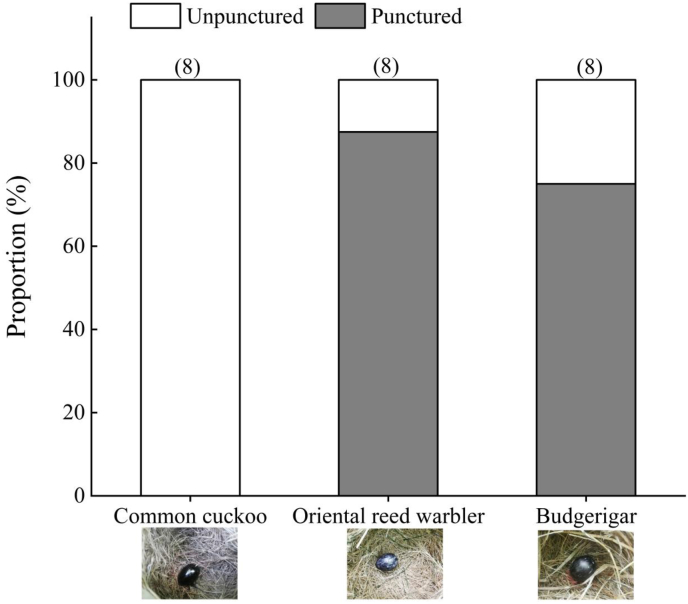
Results of the puncture egg experiment (the numbers on the figure pillars indicate the total number of eggs tested).

The results of the egg pecking experiments showed that blacked-common cuckoo eggs had 0 punctured nests (0%, n = 8 nests) compared with blacked-Oriental reed warbler eggs were punctured (87.5%, n = 8 nests), and the blacked-budgerigar eggs were punctured (75%, n = 8 nests) showing a significant difference (*P* = 0.001 and 0.007, respectively, Fisher's exact test) (Fig. 2). Punctured blacked-Oriental reed warbler eggs and blacked-budgerigar eggs did not significantly differ (Fisher's exact test: *P* = 1.000). Kruskal-Wallis test showed the number of egg pecks (*Z* = 1.654, *P* = 0.437), time of pecking (*Z* = 0.781, *P* = 0.677), and frequency (*Z* = 1.708, *P* = 0.426) between the two types of eggs did not significantly differ (Table 1). In other words, the Oriental reed warbler was able to peck through most of the blacked-colored Oriental reed warbler eggs (Video S1) and some of the blacked-colored budgerigar eggs with nearly the same pecking frequency, but was unsuccessful in pecking through all the blacked-colored cuckoo eggs (Video S2) used in the experiment.

**Table 1:** Results of the correlation parameters of Oriental reed warbler egg pecking experiments with Kruskal-Wallis test (*P* < 0.05 indicates statistical significance and SD refers to standard deviation).

	Blacked host egg (n = 7 nests)		Blacked cuckoo egg (n = 8 nests)		Blacked budgerigar egg (n = 8 nests)		*P*
	Range	Mean ± SD	Range	Mean ± SD	Range	Mean ± SD	
Number of egg pecking (times)	3–169	46.14 ± 60.19	5–716	136.13 ± 237.55	4–443	95.13 ± 147.35	0.437
Egg pecking time (second)	1–61	22 ± 21.31	1–253	54 ± 82.52	2–152	39.13 ± 49.87	0.677
Egg pecking frequency (times/second)	0.82–4	2.04 ± 1.04	1.61–5	2.55 ± 1.13	1.35-3.29	2.07 ± 0.71	0.426

## 4 Discussion

Our results indicate that Oriental reed warblers, acting as hosts of common cuckoos, exhibited almost no change in their egg-pecking frequency when faced with three different types of eggs, namely the Oriental reed warbler egg, common cuckoo egg, and budgerigar egg. This suggests that Oriental reed warblers did not significantly alter their egg-pecking frequency in response to the continuous presence of these three types of eggs. This suggests that, of the three types of eggs, Oriental reed warbler eggs were more likely to be pecked through by the Oriental reed warbler than the other two, with common cuckoo eggs being the least likely to be pecked through. This also meant that in the natural reproduction of Oriental reed warblers, when both common cuckoo eggs and Oriental reed warbler eggs are present in the Oriental reed warbler nest, the Oriental reed warbler is more likely to peck through its own eggs than the common cuckoo eggs with almost the same pecking frequency.

In brood parasitic systems, thick eggshells are often considered an adaptation to a parasitic lifestyle (Antonov et al., 2012; López et al., 2018, 2023), because hosts with smaller beaks are essentially unable to grasp the parasite's eggs with their own beaks, so the host must remove them by puncturing the parasite's eggs (Sealy 1996). Thick eggshells can prevent egg puncture in some smaller cuckoo hosts (Antonov et al., 2006, 2009; Li et al., 2016). For example, Spaw and Rohwer (1987) “tentatively” concluded that thicker eggshells were not evolutionarily essential for protection during brooding, preferring to argue that the evolution of thicker eggshells was essential for withstanding host puncture-ejection. This is consistent with our results that the eggshells of common cuckoo eggs are better able to withstand puncture-ejection by Oriental reed warblers compared to host Oriental reed warbler eggs. In addition, one of the hypotheses that has been proposed for the thick eggshell is that it resists host puncture-ejection (Swynnerton 1918; Spaw and Rohwer 1987). For example, the findings of Spottiswoode (2010) support the idea that the solid, thick-shelled eggs of the brood parasites are an adaptation that reduces the host's rejection response. Our findings also support the puncture resistance hypothesis.

The eggshell strength of avian obligate brood parasites is higher than that of their respective hosts (López et al., 2021), which have focused on eggshell thickness, egg shape or heteromorphic deviations (Spaw and Rohwer 1987; Brooker and Brooker 1991; Picman and Pribil 1997; Spottiswoode and Colebrook-Robjent 2007; Spottiswoode, 2010; McClelland et al., 2023), microhardness (Igic et al., 2011), or microstructure (Soler et al., 2019; López et al., 2023). Furthermore, Spottiswoode (2010) reported on the eggshell strength of common cuckoo eggs compared to that of other host eggs. However, to date, there have been no studies reporting the thickness of common cuckoo eggs compared to that of Oriental reed warbler eggs in China. We used experiments to validate the puncture resistance hypothesis for the first time in a cuckoo host.

Ma and Liang (2021) found that all cases of egg rejection by the Oriental reed warbler were carried out by the female and that the egg rejection rate (especially the ejection rate) of the Oriental reed warbler would show a decreasing trend in the later stages of its incubation (Li et al., 2020; but see Ma et al., 2024). Furthermore, during the nestling period, the female birds would be busy feeding the nestlings, reducing the investment costs and risky antiparasitic behaviors. This indicates that the parent birds reduce their frequency of visiting neighboring nests and their sensitivity to foreign objects in the nest. This may explain the phenomenon that some Oriental reed warblers in our experiment did not visit nests or visited nests without pecking at the eggs.

In conclusion, our findings indicate that common cuckoo eggs, as brood parasites, are less susceptible to being pecked through by host Oriental reed warblers compared to host own eggs. This suggests that, to some extent, the eggshells of common cuckoo eggs can resist the puncture-ejection behavior of the host Oriental reed warblers. Thus, we provide experimental support for the ‘puncture resistance’ hypothesis. However, we cannot deny that Oriental reed warblers can eventually destroy common cuckoo eggs laid in their nests by investing more time in the process. Although our sample size was small, our newly obtained data, to some extent, aiding in furthering our understanding of the coevolutionary dynamics in avian brood parasitism. We encourage further comprehensive research on this experiment. We suggest that future studies include experiments during the incubation period to compare and observe whether Oriental reed warblers exhibit changes in nest-visiting frequency or egg-pecking frequency between different stages of reproduction (incubation period vs. nestling period), as well as whether they are more likely to break eggs during these stages. Moreover, under ethical considerations, increasing the sample size and enhancing the accuracy of experimental validation by examining a large number of other cuckoo species' eggs and other hosts’ eggs for eggshell thickness would be beneficial.

## CRediT authorship contribution statement

**Hanlin Yan:** Writing – original draft, Investigation, Formal analysis. **Longwu Wang:** Writing – review & editing, Supervision, Conceptualization. **Wei Liang:** Writing – review & editing, Validation, Supervision, Funding acquisition, Conceptualization.

## Ethical standards

The study was conducted in compliance with the law of China. Experimental procedures in China were in accordance with the Animal Research Ethics Committee of Hainan Provincial Education Centre for Ecology and Environment, Hainan Normal University (no. HNECEE-2012-003) and Guizhou Normal University (No. GZNUECEE-2021-001).

## Data accessibility

Data used for this study and video examples were provided as supplementary material (Table S1-S2 and Video S1-S2) and can be found at https://figshare.com/s/43ea128a8e9203fc5895 (https://doi.org/10.6084/m9.figshare.27216681).

## Funding

This work was supported by the 10.13039/501100001809National Natural Science Foundation of China (Nos. 32270526 to WL, and 32260253 to LW).

## Declaration of competing interest

The authors declare that they have no known competing financial interests or personal relationships that could have appeared to influence the work reported in this paper.
